# Supplementary material for: Award rate inequities in biomedical research
Source: PLoS One. 2022 Jul 1;17(7):e0270612. doi: 10.1371/journal.pone.0270612 (PMC9249172; doi:10.1371/journal.pone.0270612)
Supplement: S1 Table — (DOCX) [file pone.0270612.s001.docx]

S1 TABLE

|  | R01/Equivalent | Other Federal | Industry | Non-Profit |
| --- | --- | --- | --- | --- |
| Asian | 28.20% | 23.90% | 72.16% | 30.62% |
| White | 29.78% | 32.46% | 72.73% | 36.66% |
| Ratio | -0.053 | -0.264 | -0.007 | -0.165 |
| Impact | Negative | Negative | Negative | Negative |
