# Supplementary material for: Award rate inequities in biomedical research
Source: PLoS One. 2022 Jul 1;17(7):e0270612. doi: 10.1371/journal.pone.0270612 (PMC9249172; doi:10.1371/journal.pone.0270612)
Supplement: S2 Table — (DOCX) [file pone.0270612.s002.docx]

S2 TABLE

|  | R01/Equivalent | Other Federal | Industry | Non-Profit |
| --- | --- | --- | --- | --- |
| B/AA | 23.43% | 19.35% | 61.11% | 33.00% |
| White | 29.78% | 32.46% | 72.73% | 36.66% |
| Ratio | -0.213 | -0.403 | -0.159 | -0.099 |
| Impact | Negative | Negative | Negative | Negative |
