# Supplementary material for: Award rate inequities in biomedical research
Source: PLoS One. 2022 Jul 1;17(7):e0270612. doi: 10.1371/journal.pone.0270612 (PMC9249172; doi:10.1371/journal.pone.0270612)
Supplement: S3 Table — (DOCX) [file pone.0270612.s003.docx]

S3 TABLE

|  | R01/Equivalent | Other Federal | Industry | Non-Profit |
| --- | --- | --- | --- | --- |
| Hispanic/Latino | 37.32% | 28.47% | 75.75% | 43.57% |
| White | 29.78% | 32.46% | 72.73% | 36.66% |
| Ratio | 0.253 | -0.122 | 0.041 | 0.188 |
| Impact | Positive | Negative | Positive | Positive |
