# Supplementary material for: Award rate inequities in biomedical research
Source: PLoS One. 2022 Jul 1;17(7):e0270612. doi: 10.1371/journal.pone.0270612 (PMC9249172; doi:10.1371/journal.pone.0270612)
Supplement: S4 Table — (DOCX) [file pone.0270612.s004.docx]

S4 TABLE

|  | R01/Equivalent | Other Federal | Industry | Non-Profit |
| --- | --- | --- | --- | --- |
| Asian | 31.18% | 28.16% | 7.04% | 33.60% |
| White | 30.17% | 28.96% | 8.14% | 32.72% |
| Ratio | 0.033 | -0.028 | -0.135 | 0.027 |
