# Supplementary material for: Award rate inequities in biomedical research
Source: PLoS One. 2022 Jul 1;17(7):e0270612. doi: 10.1371/journal.pone.0270612 (PMC9249172; doi:10.1371/journal.pone.0270612)
Supplement: S5 Table — (DOCX) [file pone.0270612.s005.docx]

S5 TABLE

|  | R01/Equivalent | Other Federal | Industry | Non-Profit |
| --- | --- | --- | --- | --- |
| B/AA | 26.22% | 25.40% | 7.37% | 40.98% |
| White | 30.17% | 28.96% | 8.14% | 32.72% |
| Ratio | -0.131 | -0.123 | -0.095 | 0.252 |
