# Supplementary material for: Award rate inequities in biomedical research
Source: PLoS One. 2022 Jul 1;17(7):e0270612. doi: 10.1371/journal.pone.0270612 (PMC9249172; doi:10.1371/journal.pone.0270612)
Supplement: S6 Table — (DOCX) [file pone.0270612.s006.docx]

S6 TABLE

|  | R01/Equivalent | Other Federal | Industry | Non-Profit |
| --- | --- | --- | --- | --- |
| Hispanic/Latino | 28.11% | 29.90% | 6.53% | 35.44% |
| White | 30.17% | 28.96% | 8.14% | 32.72% |
| Ratio | -0.068 | 0.032 | -0.198 | 0.083 |
