# Supplementary material for: Award rate inequities in biomedical research
Source: PLoS One. 2022 Jul 1;17(7):e0270612. doi: 10.1371/journal.pone.0270612 (PMC9249172; doi:10.1371/journal.pone.0270612)
Supplement: S7 Table — The award and submission ratios for each racial/ethnic group-submission category pair. (DOCX) [file pone.0270612.s007.docx]

S7 TABLE

|  | **R01 /Equivalent** | | **Other Federal** | **Industry** | **Non-Profit** |  |  |  |
| --- | --- | --- | --- | --- | --- | --- | --- | --- |
| **Asian** | | + - | - - | - - | + - |  | + + | Submission and Award ratios are both positive relative to majority |
| **B/AA** | | - - | - - | - - | + - |  | + - | Submission ratio is positive and Award ratio is negative relative to majority |
| **Hispanic/Latino** | | - + | + - | - + | + + |  | - - | Submission and Award ratios are both negative relative to majority |
|  |  | |  |  |  |  | - + | Submission ratio is negative and Award ratio is positive relative to majority |
